# Supplementary material for: Power generation by a myosin-1 essential for endocytosis: implications for biological mechanism
Source: bioRxiv. 2023 May 17:2023.03.21.533689. Originally published 2023 Mar 22. Preprint. [Version 2] doi: 10.1101/2023.03.21.533689 (PMC10055380; doi:10.1101/2023.03.21.533689)
Supplement: Supplementary file 1 [file media-1.pdf]

Figure S1

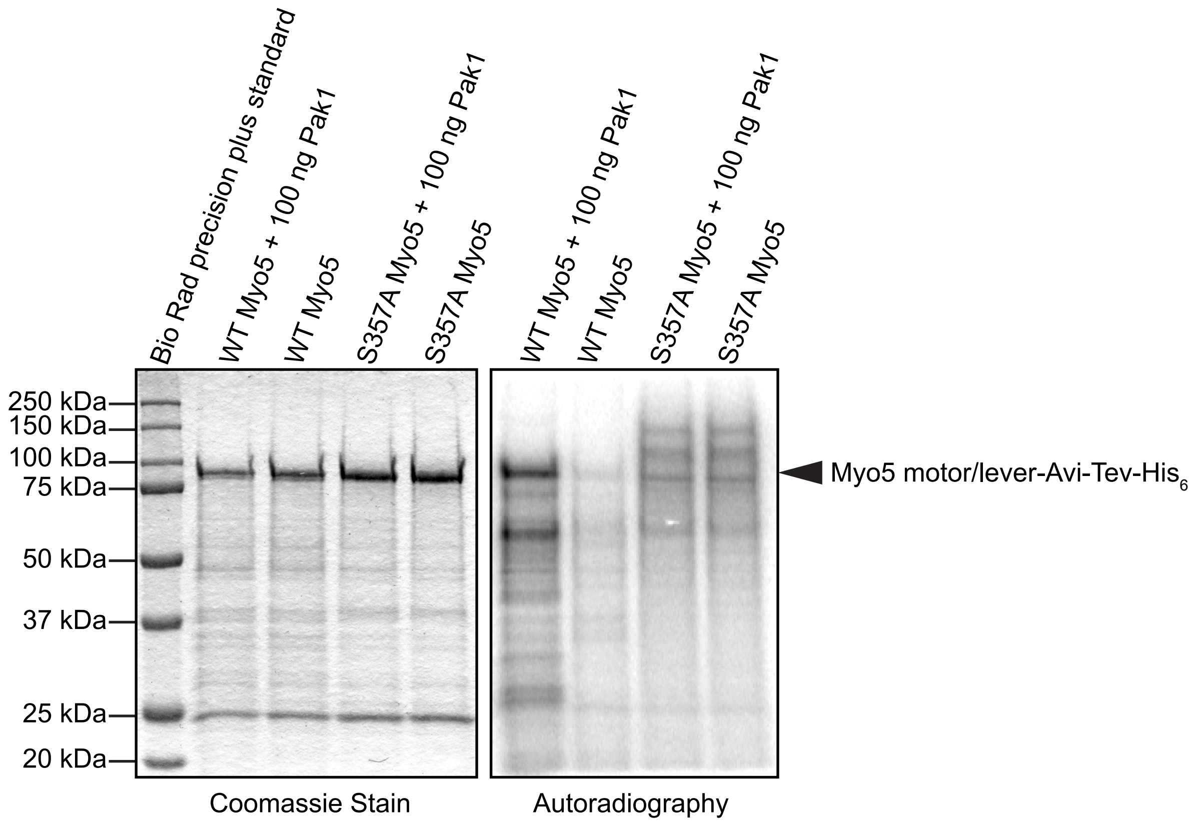

Bio Rad precision plus standard
WT Myo5 + 100 ng Pak1
WT Myo5
S357A Myo5 + 100 ng Pak1
S357A Myo5
WT Myo5 + 100 ng Pak1
WT Myo5
S357A Myo5 + 100 ng Pak1
S357A Myo5
250 kDa
150 kDa
100 kDa
75 kDa
50 kDa
37 kDa
25 kDa
20 kDa
Myo5 motor/lever-Avi-Tev-His$_6$
Coomassie Stain
Autoradiography
